# Impact of sitagliptin combination therapy and hypoglycemia in Japanese patients with type 2 diabetes: a multi-center retrospective observational cohort study

**DOI:** 10.1186/s40780-020-00169-5

**Published:** 2020-06-04

**Authors:** Tomoyuki Saito, Hirotoshi Ohmura, Shuko Nojiri, Hiroyuki Daida

**Affiliations:** 1 Department of Cardiovascular Medicine, Juntendo University Graduate School of Medicine, 2-1-1 Hongo, Bunkyo-Ku, Tokyo, 113-8421 Japan; 2 Department of Pharmacy, Juntendo University Hospital, 3-1-3 Hongo, Bunkyo-Ku, Tokyo, 113-8431 Japan; 3 Juntendo University, Medical Technology Innovation Center, 3-1-3 Hongo, Bunkyo-Ku, Tokyo, 113-8431 Japan

**Keywords:** Hypoglycemia, Dipeptidyl-peptidase IV inhibitors, Beta-blockers,, Type 2 diabetes mellitus

## Abstract

**Background:**

Patients with diabetes are at higher risk of developing polypharmacy because of the high frequency of comorbidities. There have been several reports on the hypoglycemic risk of the combination of hypoglycemic agents and other medications. This study aimed to investigate the hypoglycemic risk of drug-drug interaction between sitagliptin and other oral hypoglycemic agents or antihypertensive agents in Japanese patients with type 2 diabetes.

**Methods:**

From January 2010 to March 2012, a total of 3247 patients were recruited and evaluated at outpatient clinics at Juntendo University Hospital, other satellite hospitals, and private clinics. This study was a sub-analysis of the Sitagliptin Registration Type 2 Diabetes-Juntendo Collaborating Project. Participants were limited to those treated with oral hypoglycemic agents, excluding insulin users, to investigate the association of the first hypoglycemic events with oral hypoglycemic agents or other medications within 6 months after starting sitagliptin. The factors related to the first hypoglycemic event were analyzed using Cox regression analysis.

**Results:**

In total, 2956 patients with a mean age of 65.1 ± 11.3 years were included. A total of 46 hypoglycemic events (1.6%) were observed. One patient had severe hypoglycemia followed by emergency transport to the hospital. Sitagliptin was not associated with hypoglycemia, but its combination with sulfonylurea (hazard ratio: 4.42, 95% confidential interval: 1.36–14.42) or β-blocker (hazard ratio, 3.50, 95% confidential interval: 1.54–7.96) was significantly associated with hypoglycemia.

**Conclusions:**

The drug-drug interactions between sitagliptin and sulfonylurea or β-blocker likely increases the hypoglycemic risk in Japanese patients with type 2 diabetes. Pharmacists should consider potential adverse events from drug-drug interaction in type 2 diabetes with polypharmacy, particularly those who are managed by several doctors or clinics.

## Background

The prevalence of type 2 diabetes (T2DM) has been markedly increasing worldwide in recent decades [1]. In Japan, approximately 10 million people have T2DM, and it is estimated that just as many people potentially have T2DM [2]. T2DM is associated with reduced quality of life (QOL) and shortened life expectancy associated with microvascular complications [3–9]. Therefore, the ultimate goals of T2DM management are to maintain QOL and to prolong life expectancy by preventing vascular complications [10].

It has been reported that maintaining good glycemic control reduces the risk of microvascular complications [11–14]. However, previous clinical trials have failed to show the effects of intensive glycemic control on reducing the incidence of macrovascular complications [12, 14–16]. According to the Action to Control Cardiovascular Risk in Diabetes (ACCORD) trial, intensive glucose-lowering therapy failed to reduce major cardiovascular events. Moreover, severe hypoglycemia with intensive therapy resulted in increased mortality [17]. Therefore, the prevention of hypoglycemia is an important issue in the treatment of diabetes.

Patients with T2DM are more likely to have metabolic disorders such as hypertension, dyslipidemia, underlying insulin resistance, and other comorbidities. However, optimal comprehensive risk management including glucose, lipid, and blood pressure control demonstrated beneficial effects on decreasing the incidence of both microvascular and macrovascular complications and even on all-cause mortality [18–20]. Consequently, these patients are generally treated with many pharmacological compounds, commonly referred as “polypharmacy” [21, 22]. With this type of treatment, patients generally are at a high risk for drug-drug interactions. Polypharmacy is one of the risk factors associated with hypoglycemia as well as a lower education level, ethnicity, irregular meals/malnutrition, insulin and sulfonylurea therapy, cognitive impairment, depression and frailty. Among patients with T2DM, diabetic complications are more frequent in those with hypertension than in those without hypertension [7, 23–25]. However, a few antihypertensive agents have also been associated with an increased incidence of hypoglycemia and an increased risk of cardiovascular events [26]. Therefore, it is necessary to clarify the effects of the combined use of each OHA and antihypertensive drugs on hypoglycemia within a clinical setting.

Thus, the present study aimed to investigate the risk of hypoglycemia related to drug-drug interaction between OHAs and antihypertensive agents in Japanese patients with T2DM as a sub-analysis from the Sitagliptin Registration Type 2 Diabetes-Juntendo Collaborating Project (SPIRITS-J).

## Methods

### Setting and study cohort

**Fig. 1:**
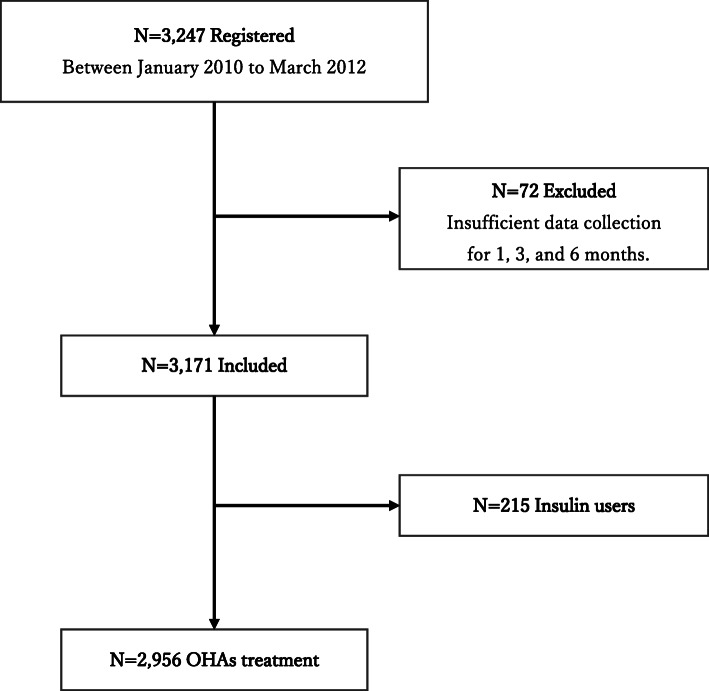
Patient selection flow diagram in the observational cohort study. OHAs, oral hypoglycemic agents

The SPIRITS-J study was a multi-center retrospective observational cohort study designed to evaluate the efficacy and safety of sitagliptin supplementation for over 6 months. Participants were Japanese patients with T2DM who were treated with diet and exercise, other OHA combination therapy, and/or insulin therapy. All procedures were conducted according to the 1964 Declaration of Helsinki revised in 2013 and the Ethical Standards of the Responsible Committee on Human Experimentation. From January 2010 to March 2012, a total of 3247 patients were recruited and evaluated at outpatient clinics at Juntendo University Hospital, other satellite hospitals, and private clinics. We conducted a sub-group analysis in 2956 patients with T2DM who were excluded from insulin treatment (Fig. 1). In a study conducted by the Japanese Society of Glucose and Diabetes to investigate severe hypoglycemia, insulin was responsible for approximately 60% of severe hypoglycemia in patients with T2DM [15]. Hypoglycemia was excluded from the analysis because it was significantly affected by insulin treatment. This study was enrolled in the UMINS clinical trial registry (UMIN 000004121).

### Data collection

Data were collected at four time points within 6 months after the initiation of sitagliptin (baseline, 1, 3, and 6 months). Baseline clinicodemographic data of all the patients enrolled in this study were collected, including age, sex, body mass index (BMI), diabetes duration, diabetes-related complications, medical history, lipid profile, random blood glucose, HbA1c, serum creatinine, and ingredient of medication for hypertension and diabetes. Blood test results were obtained from the medical records. Glucose and HbA1c were measured with standard techniques. The value of HbA1c (%) was estimated as the National Glycohemoglobin Standardization Program (NGSP) equivalent value (%) calculated using the formula HbA1c (%) = HbA1c (Japan Diabetes Society) (%) + 0.4% [16]. The estimated glomerular filtration rate (eGFR) was calculated using the following formula: eGFR (mL/min/1.73 m^2^) = 194 × age − 0.287 × serum creatinine − 0.1094 (× 0.739 for women) [27].

### Definition of hypoglycemia

We collected information on the first hypoglycemic event from patient self-report, medical records, and blood analysis from medical examinations with a blood glucose level of ≤60 mg/dL. Therefore, it includes subjective symptoms determined to be caused by hypoglycemia regardless of blood glucose level. The participants did not conduct self-monitoring of blood glucose (SMBG) because in the Japanese healthcare system, SMBG is covered by insurance only in insulin users. Severe hypoglycemia represented hypoglycemic episodes that required the attention and assistance of another person.

### Definition of diabetic microvascular complications

Retinopathy was defined as a progression from the absence of retinopathy to nonproliferative retinopathy or proliferative retinopathy, progression from nonproliferative retinopathy to proliferative retinopathy, or loss of vision due to retinopathy. Nephropathy is the progression from normal albuminuria to macroalbuminuria or from microalbuminuria to macroalbuminuria; an increase in serum creatinine concentration at least twice the study baseline; or end-stage renal failure, neuropathy, or neuropathy unrelated to peripheral neuropathy or spinal stenosis.

### Statistical analysis

Results are presented as mean ± standard deviation (SD) or percentage (%). The HbA1c values observed at the four time points were calculated using a mixed-effects model to account for repeated measurements of participants and the random effects of observations. *P*-value was calculated using type III tests of fixed effects. Differences between baseline data and any observation points were examined for statistical significance using the Student’s t-test. Categorical variables were compared using the Chi-squared test and presented as absolute frequencies with percentages. The factors associated with hypoglycemic events were examined using Cox Regression analysis. A two-sided *P*-value of < 0.05 was considered statistically significant. The hazard ratio (HR) in cohort studies is positively correlated to the cumulative disease incidence [28]. Hence, it is important that enough events occur during the analysis period. In our previous study, we reported that blood glucose levels decreased significantly during the first month after the start of treatment with sitagliptin [6]. In addition, it has been found that hypoglycemia tends to increase during that period. Therefore, the Cox regression model was used considering the dynamic characteristics of the expected distribution. Moreover, it was confirmed that the proportional hazards in the Cox regression model were appropriate. All analyses were performed using SAS Ver. 9.4 (SAS Institute Inc., Cary, NC, USA).

## Results

### Baseline clinicodemographic characteristics

Table 1 demonstrates the baseline clinicodemographic characteristics of the study patients. The mean T2DM duration was 10.2 ± 7.5 years, and the mean HbA1c level was 7.8 ± 1.2%. The mean age was 65.1 ± 11.3 years. In total, 459 (15.6%) patients had a history of coronary artery disease, and only approximately 15% of patients had at least one diabetic microvascular complication.

**Table 1:** Baseline characteristics of the study subjects

	OHA patients (*n* = 2956)	non-hypoglycemia (*N* = 2910)	hypoglycemia (*N* = 46)	*P*-value
Sex (male%)	1772 (59.9%)	1749 (60.1%)	23 (50.0%)	0.1653
Age	65.1 ± 11.3	65.1 ± 11.3	65.9 ± 8.6	0.5311
BMI^a^	25.0 ± 4.3	25.1 ± 4.3	23.5 ± 3.9	**0.0228**
Smoking status				0.6357
Current users	614 (23.5%)	603 (23.5%)	11 (26.8%)	
Past users	531 (20.3%)	525 (20.5%)	6 (14.6%)	
Never users	1463 (56.1%)	1439 (56.1%)	24 (58.5%)	
Diabetic duration	10.2 ± 7.5	10.2 ± 7.5	12.4 ± 7.2	0.0784
Comorbidity				
Coronary artery diseases	9 (0.3%)	9 (0.3%)	0 (0.0%)	0.7081
Cerebrovascular diseases	276 (9.4%)	273 (9.4%)	3 (6.7%)	0.5295
Diabetic microvascular complications				
Retinopathy	418 (14.2%)	413 (14.3%)	5 (11.1%)	0.5493
Neuropathy	345 (11.7%)	339 (11.7%)	6 (13.3%)	0.7350
Nephropathy	514 (17.4%)	508 (17.5%)	6 (13.3%)	0.4619
Medication				
Number of combined OHA	1.3 ± 1.0	1.3 ± 1.0	2.1 ± 1.0	**<.0001**
Clinical data				
HbA1c (NGSP) (%)	7.8 ± 1.2	7.8 ± 1.2	7.9 ± 1.2	0.4905
eGFR^b^	76.6 ± 22.9	76.7 ± 22.9	72.7 ± 22.6	0.2649
HDL-C	55.6 ± 15.0	55.5 ± 15.0	56.2 ± 14.9	0.8018
TC	193.8 ± 37.1	193.9 ± 37.2	190.4 ± 27.6	0.5306
TG	155.0 ± 127.2	155.2 ± 127.8	140.8 ± 87.5	0.3002
Uric acid	5.2 ± 1.6	5.2 ± 1.6	4.6 ± 1.4	**0.0325**

*BMI* Body-mass index, *OHAs* Oral hypoglycemic agents, *eGFR* Estimated glomerular filtration rate, *HDL-C* High-density lipoprotein cholesterol, *TC* Total cholesterol, *TG* Triglyceride

^a^The body-mass index is body weight in kilograms divided by the square of height in meters

^b^The estimated GFR was calculated using the modified Modification of Diet in Renal Disease (MDRD) formula

Table 2 demonstrates the usage of OHAs and anti-hypertensive agents in this study. Overall, 764 (25.5%) patients had treatment with sitagliptin monotherapy. The most common class of OHAs combined with sitagliptin was sulfonylurea (SU) (50.4%), followed by biguanide (39.5%), thiazolidinedione (TZD) (22.7%), alpha-glucosidase inhibitors (α-GI) (13.5%), and glinides (2.0%). For the antihypertensive agents, angiotensin-converting enzyme inhibitors/angiotensin II receptor blockers (59.3%) and calcium channel blockers (46.9%) were the most commonly prescribed in this study. Meanwhile, β-blockers (BB) (13.6%) and diuretics (9.7%) was less frequently prescribed.

**Table 2:** Use of oral antidiabetic agents in the study subjects stratified by hypoglycemic event

Drugs	Overall population	Non-hypoglycemia	Hypoglycemia	*P*-value*
	N (%)	N (%)	N (%)	
SU	1468 (50.4)	1428 (49.8)	40 (87.0)	**< 0.0001**
TZD	662 (22.7)	646 (22.5)	16 (34.8)	**0.0490**
Glinide	59 (2.0)	59 (2.1)	0 (0.0)	
AGI	393 (13.5)	378 (13.2)	15 (32.6)	**0.0001**
Biguanide	1152 (39.5)	1125 (39.2)	27 (58.7)	**0.0074**
β-blocker	318 (13.6)	307 (13.4)	11 (27.5)	**0.0098**
Diuretic	226 (9.7)	222 (9.7)	4 (10.0)	0.9440
ACE/ARB	1384 (59.3)	1362 (59.3)	22 (55.0)	0.5814
CCB	1096 (46.9)	1079 (47.0)	17 (42.5)	0.5723

*SU* Sulfonylurea, *TZD* Thiazolidinedione, *AGI* Alpha-glucosidase inhibitors, *ACE-I* Angiotensin-converting enzyme inhibitors, *ARB* Angiotensin II receptor blocker, *CCB* Calcium channel blocker

*Chi-squared tests were used in the analyses of categorical variables

### Characteristics of the patients with hypoglycemia

A total of 46 hypoglycemic events (1.6%) were observed during the first 6 months after starting sitagliptin. One patient had severe hypoglycemia followed by emergency transport to the hospital. The patients with hypoglycemic events had a significantly higher number of combined OHAs (2.1 ± 1.0 vs. 1.3 ± 1.0, *p* < 0.0001) and significantly lower BMI (23.5 ± 3.9 vs. 25.1 ± 4.3, *p* = 0.0228) and plasma levels of UA (4.6 ± 1.4 vs. 5.2 ± 1.6, *p* = 0.0325) (Table 1). There was no significant difference in the duration of T2DM, although it tended to be longer in patients with hypoglycemic events (12.4 ± 7.2 vs. 10.2 ± 7.5, *p* = 0.0784). In addition, the prevalence of macrovascular and microvascular complications was not significantly different between the two groups. Regarding the OHAs and anti-hypertensive agents, the usage of SU, TZD, α-GI, biguanide, and BB was significantly higher in patients with hypoglycemia.

In multivariate Cox regression model for the risk of hypoglycemia adjusted for sex, age, BMI, prescription number of OHAs, and eGFR, low BMI (HR: 0.86, 95% confidential interval (CI): 0.78–0.95), low uric acid (HR: 0.67, 95% CI: 0.49–0.90), use of SU (HR: 4.42, 95% CI: 1.36–14.42), and BB (HR: 3.50, 95% CI: 1.54–7.96) remained to be independent risk factors for hypoglycemia (Table 3).

**Table 3:** Multivariate Cox regression model for hypoglycemia risk

	No. of cases	Person-years	Incidence rate per 100 population	HR	95% CI	*P*-value
Sitagliptin						
monotherapy	3	378	0.8	1.00		
+ SU	40	718	5.6	4.42	(1.36–14.42)	**0.014**
+ TZD	16	326	4.9	0.55	(0.23–1.34)	0.189
+ GLINIDE	0	29	0.0			
+ AGI	15	193	7.8	0.90	(0.37–2.22)	0.820
+ BIGUANIDE	27	566	4.8	0.83	(0.35–2.01)	0.685
Antihypertensive agents						
+ β-blocker	11	157	7.0	3.50	(1.54–7.96)	**0.003**
+ Diuretic	4	111	3.6	1.67	(0.56–4.96)	0.359
+ ACE/ARB	22	681	3.2	0.91	(0.42–1.97)	0.819
+ CCB	17	541	3.1	0.97	(0.45–2.11)	0.942

Adjusted for sex, age, BMI, number of combined OHAs, and eGFR

*SU* sulfonylurea, *TZD* Thiazolidinedione, *AGI* Alpha-glucosidase inhibitors, *ACE-I* Angiotensin-converting enzyme inhibitors, *ARB* Angiotensin II receptor blocker, *CCB* Calcium channel blocker, *HR* Hazard ratio, *CI* Confidence interval

## Discussion

The present study investigated the risk of hypoglycemia from drug-drug interactions between sitagliptin and other OHAs or antihypertensive agents in Japanese patients with T2DM. In the SPIRITS-J study, the incidence of hypoglycemia in sitagliptin treatment combined with other OHAs was 1.6%. This result suggested the safety of sitagliptin as monotherapy or in combination with other OHAs in Japanese patients with T2DM. However, the multivariate Cox regression model demonstrated that the use of SU or BB were independent risk factors for hypoglycemia.

We have already reported that the risk of hypoglycemia associated with DPP-IV inhibitors is low, and that the risk increased when combined with SU. However, there are not enough reports on the hypoglycemic risk associated with drug combinations other than OHAs in patients with diabetes who have many complications. In particular, we investigated the drug-drug interactions with antihypertensive agents as a sub-analysis of SPIRITS-J.

Although maintaining good glycemic control reduces the risk of microvascular complications, intensive glycemic control in the ACCORD trial resulted in increased all-cause and cardiovascular mortalities [18]. A possible explanation is that intensive glycemic control increased the risk of hypoglycemia and consequently increased the risk of major vascular events and death. Therefore, appropriate glycemic control without hypoglycemic events is important for preventing major cardiovascular events in patients with T2DM.

SU is widely used to treat T2DM because it the lowers blood glucose levels by releasing insulin from beta cells in the pancreas [29]. DPP-4 inhibitors stimulate insulin secretion, suppress glucagon secretion, and reduce gastric emptying in a glucose-dependent manner [30]. Although severe hypoglycemia has been reported even with the single usage of SU [31], a meta-analysis of combination therapy efficacy of DPP-4 inhibitors with SU in patients with T2DM showed that the risk of hypoglycemia was increased by approximately 50% [32]. Therefore, the Japan Association for Diabetes Education and Care has recommended that decreasing the SU dosage should be considered in case of concomitant use with DPP-4 inhibitors [33]. The Trial Evaluating Cardiovascular Outcomes with Sitagliptin (TECOS) study recently reported that the combination of sitagliptin and SU did not increase the risk for severe hypoglycemia [34]. In our study, only one patient had severe hypoglycemia, but the concomitant use of SU with sitagliptin was an independent risk factor for hypoglycemia.

Polypharmacy is common in patients with T2DM because they often have co-morbidities; thus, it is necessary to consider the effect of hypoglycemia from drug-drug interactions [35]. Both hypertension and coronary artery disease (CAD) are frequent comorbidities in patients with T2DM. Moreover, diabetic complications are more frequent in T2DM patients with hypertension than in T2DM patients without hypertension [24, 25]. The efficacy of BB, which is one of the more popular anti-hypertensive agents, for preventing major cardiovascular events and improving prognosis has been established in patients with CAD [36, 37]. However, the benefit of BB in patients with T2DM has been controversial because BB is known to be associated with a risk for severe hypoglycemia [38]. In a post-hoc analysis of the ACCORD study, the use of BB in patients with T2DM was associated with not only the incidence of severe hypoglycemia, but also increased risk for cardiovascular events even in patients with CAD [36, 37]. Meanwhile, in patients with T2DM and atherosclerotic cardiovascular disease enrolled in the TECOS trial, which evaluated the effect of sitagliptin on cardiovascular outcomes, BB use was not associated with a risk for severe hypoglycemia but also was not associated with reduction of cardiovascular risk [39]. A recent meta-analysis reported that BB therapy in T2DM patients with stable CHD is associated with higher mortality [40]. This is supported by our findings that BB therapy in T2DM more likely increases the risk of hypoglycemia, consequently increasing the risk of major adverse events. Again, the benefit of BB in patients with T2DM remains unclear.

Insulin, SU, and alcohol are common causes of drug-induced hypoglycemia, but hypoglycemia occurs in around 10% of cases without the use of these drugs [41]. Among the 10% of alternate causes of drug-induced hypoglycemia, diuretics, BB, sympathomimetics, corticosteroids, and sex hormones are frequently reported. Carbohydrate metabolism may be adversely affected in patients with hypoglycemia, especially in those with diabetes or impaired glucose tolerance [42]. There are frequent reports that BB affect hypoglycemia, suggesting that they affect liver and kidney function through β receptors [41].

The combination of BB and DPP-IV inhibitors increases the risk of hypoglycemia, which can be explained by two factors. First, BB has been reported to mask the symptoms of hypoglycemia by reducing peripheral antiadrenergic effects [38, 43]. However, in the ACCROD trial, BB was reported to increase hypoglycemic events [26]. In our study, hypoglycemia also increased in patients using BB. The fact that BB did not mask hypoglycemic symptoms may be related to β-receptor subtypes. Cardiac muscle has a large distribution of β1 receptors, and skeletal muscle has β3 receptors [44]. Therefore, since BBs used in recent years comprise several selective β1 receptors, it is difficult to mask hypoglycemic symptoms with the drugs. Frequently used carvedilol exhibits both nonselective β-blocking and weak α-receptor blocking effects; however, carvedilol has a weak β3 receptor blocking effect [45]. Second, selective β2-blockers suppress glycogen degradation and can counter act hypoglycemia owing to their effects on the liver and muscles [46]. Furthermore, β-receptor blockade suppresses glucagon secretion due to antiadrenergic action [47]. There is currently no clinical basis for these pharmacological actions, and clinical verification is needed in the future.

Our study has some limitations. First, this was an observational study with no randomization and may have selection bias. Therefore, the results of this study should be interpreted cautiously. Second, there was little information about lifestyle, diet and exercise therapy, drug adherence, and confounding effects of other medications. Furthermore, information on the proportion and doses of OHAs and BB at 6 months was lacking. Third, laboratory tests were not standardized throughout the study. Fourth, the type of each drug, particularly, the type of BB, could not be identified. Among BB types, non-vasodilating BB has been suggested to be associated with hyperglycemia, while vasodilating BB is associated with hypoglycemia [48]. Further prospective randomized studies are needed to elucidate the benefits and disadvantages of BB therapy in patients with T2DM.

## Conclusion

The drug-drug interaction between sitagliptin and sulfonylurea or β-blocker likely increases the hypoglycemic risk in Japanese patients with type 2 diabetes. Pharmacists should consider potential adverse events from drug-drug interactions in patients with T2DM with polypharmacy, particularly those managed by several doctors or clinics.

## Data Availability

The datasets generated during and/or analyzed during the current study are available from the corresponding author on reasonable request.

## Abbreviations

T2DM: Type 2 diabetes
QOL: Quality of life
ACCORD: Action to Control Cardiovascular Risk in Diabetes study
GLP-1: Glucagon-like peptide-1
GIP: Glucose-dependent insulinotropic polypeptide
OHAs: Oral hypoglycemic agents
HbA1c: Hemoglobin A1c
J-DOIT 3: The Japan Diabetes Optimal Integrated Treatment study
SPIRITS-J: The Sitagliptin Registration Type 2 Diabetes-Juntendo Collaborating Project
BMI: Body mass index
NGSP: The National Glycohemoglobin Standardization Program
eGFR: The estimated glomerular filtration rate
SMBG: Self-monitoring of blood glucose
SD: Standard deviation
SU: Sulfonylurea
TZD: Thiazolidinedione
α-GI: alpha-glucosidase inhibitors
BB: β-blockers
HR: Hazard ratio
TECOS: The Trial Evaluating Cardiovascular Outcomes with Sitagliptin study
CAD: Coronary artery disease
